# Fed-batch strategies for the enhanced biotransformation of *cis*-epoxysuccinate to L-( +)-tartrate

**DOI:** 10.1186/s40643-026-01010-x

**Published:** 2026-01-27

**Authors:** Jia-Jun Ouyang, Jiang Pan, Jian-He Xu, Chun-Xiu Li, Xu-Dong Kong

**Affiliations:** 1 State Key Laboratory of Bioreactor Engineering, Shanghai Collaborative Innovation Center for Biomanufacturing, East China University of Science and Technology, 130 Meilong Road, Shanghai, 200237 China; 2 State Key Laboratory of Microbial Metabolism and School of Life Sciences and Biotechnology, Shanghai Jiao Tong University, 800 Dongchuan Road, Shanghai, 200240 China; 3 Zhangjiang Institute for Advanced Study, Shanghai Jiao Tong University, 429 Zhangheng Road, Shanghai, 201203 China

**Keywords:** L-(+)-Tartaric acid, *cis-*Epoxysuccinate hydrolase, Fed-batch reaction, Cell immobilization, Space–time yield

## Abstract

L-( +)-Tartaric acid is a valuable organic acid with broad applications in the food, pharmaceutical, and chemical industries. Its eco-friendly synthesis typically relies on the enzymatic hydrolysis of *cis*-epoxysuccinate (CES) catalyzed by *cis*-epoxysuccinate hydrolases (CESHs), but conventional single-batch processes suffer from low space–time yields and poor continuity. To address these challenges, we devised two complementary fed-batch strategies to simplify the enzyme–product separation by exploiting differences in their solubilities. Strategy A employs carrier-free cross-linking immobilization of whole cells using 0.02% glutaraldehyde and 0.1% polyethylenimine. In this system, both the substrate sodium *cis*-epoxysuccinate (CESNa) and the product sodium L-( +)-tartrate remain soluble, while the enzyme is retained in the insoluble cell matrix. Under fed-batch operation, this configuration achieves a space–time yield of 150 g L^−1^ h^−1^. Strategy B uses cell-free extract of CESH to hydrolyze calcium *cis*-epoxysuccinate (CESCa) with inherently low solubility. Here, the enzyme is fully soluble but the L-( +)-tartrate formed precipitates as an insoluble calcium salt, allowing easy separation of the product from the reaction mixture. This approach overcomes potential substrate inhibition and minimizes sodium-ion discharge, delivering a space–time yield of 136 g L^−1^ h^−1^ and a specific productivity of 484 g_product_/g_catalyst_. Both the soluble-product/insoluble-enzyme system (A) and the insoluble-product/soluble-enzyme system (B) represent effective strategies to streamline downstream processing and markedly enhance productivity. Together, they offer a viable route to scalable and cost-effective industrial production of L-( +)-tartaric acid.

**Graphical Abstract:**

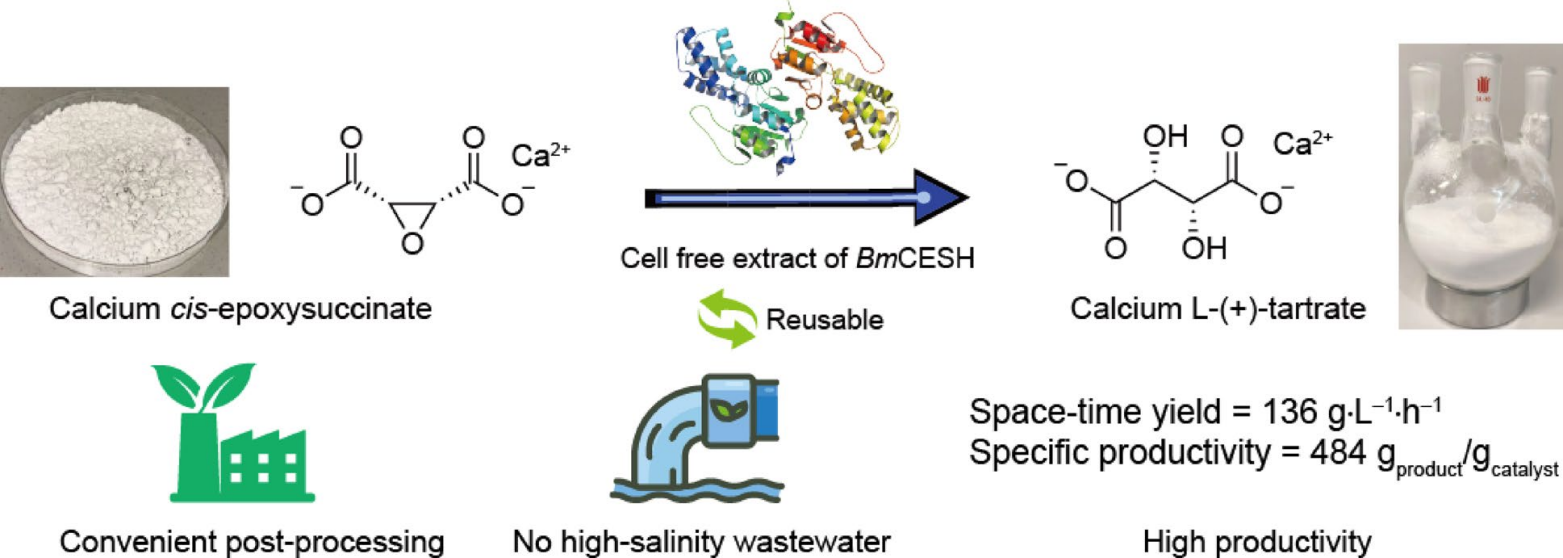

## Introduction

L-( +)-Tartaric acid (L-TA) is a naturally occurring chiral dicarboxylic acid that finds widespread application in the pharmaceutical, materials-science and food industries (Serra et al. 2005). In pharmaceutical synthesis, L-TA is routinely employed as a cost-effective resolving agent to afford enantiomerically pure active pharmaceutical ingredients such as Vigabatrin (Karumanchi et al. 2018; Sobiecka et al. 2017). In materials science, its bifunctional nature, as both a plasticizer and a model substrate for thermal and optical characterization, underscores its versatility in polymer and device research (Dong et al. 2023; Thirupathy 2022). Within the food industry, L-TA is widely used as an acidulant in winemaking and as a pH regulator in baking applications (Zhao et al. 2023; Lo Faro et al. 2024). Conventional extraction of L-TA from wine lees (potassium bitartrate) is inadequate to satisfy growing global demand. Accordingly, its industrial production now relies primarily on *cis*-epoxysuccinate hydrolase (CESH), which stereospecifically hydrolyzes *cis*-epoxysuccinic acid to L-TA (with CESH[L]) or D-TA (with CESH[D]) under mild, cofactor- and metal-ion-free conditions, delivering product with ≥ 99% enantiomeric excess (Pan et al. 2010).

Due to its exceptional enantioselectivity and outstanding catalytic efficiency, CESH has attracted extensive academic investigation and has been implemented in numerous industrial production processes. Furthermore, CESH[L] has been identified in diverse bacterial genera (Xuan and Feng 2019). Genes from *Rhodococcus opacus* (*Ro*CESH, ABF01020), *Nocardia tartaricans* CAS-52 (*Nt*CESH, AFE86013), and *Klebsiella oxytoca* BK-58 (*Ko*CESH, AHL19851) have been recombinantly expressed in *Escherichia coli* (Liu et al. 2007; Wang et al. 2013; Cheng et al. 2014). Site-directed mutagenesis has identified catalytically essential residues, revealing a two-step ring-opening mechanism (Vasu et al. 2012). More recently, crystallographic studies revealed an Asp–His–Glu catalytic triad and an arginine residue that protonates the oxirane oxygen to effect stereoselective epoxide cleavage (Dong et al. 2024; Han et al. 2024). These findings not only provide fundamental insights into the mechanistic understanding of CESH[L], but also establish a theoretical framework for identifying more efficient CESH[L] homologs through genome mining (Han et al. 2024).

From an industrial perspective, up-scaling of L-TA production hinges on three key cost determinants: biocatalyst preparation, substrate acquisition, and downstream purification. To mitigate catalyst expenses, cell- or enzyme-immobilization techniques have been developed to enable catalyst reuse and eliminate repeated fermentation cycles. For instance, carrier-based approaches such as polyester nonwoven fabric-immobilized bacterial cells (Zhang et al. 2024) and particle-anchored β-xylosidase (Vasquez et al. 2025) showed improved stability in respective applications. κ-Carrageenan–entrapped *Labrys* sp. BK-8 cells (Bao et al. 2015) and cellulose-binding CBM30 fusions (Wang et al. 2012) have shown improved operational stability in L-TA production, albeit at the expense of additional support materials. In contrast, carrier-free immobilization (e.g., cross-linked cell aggregates) achieves stabilization of the biocatalyst without exogenous scaffolds. In the context of L-phosphinothricin biosynthesis, this strategy delivered ten successive reuse cycles and extended the enzyme half-life by 10.8-fold at elevated temperatures (Zou et al. 2022).

Despite these advances in reducing catalyst-related costs, significant challenges remain in substrate handling and downstream separation of product and biocatalyst. The widely employed sodium *cis*-epoxysuccinate (CESNa) substrate system, for example, necessitates laborious calcium salt precipitation during downstream product extraction and generates high-salinity effluent streams that complicate wastewater treatment (Miková. et al. 1998). Direct use of calcium *cis*-epoxysuccinate (CESCa) as the substrate obviates the need for calcium salt precipitation during L-TA extraction and avoids sodium-ion introduction, yielding both environmental and cost benefits. However, as reported by Wang et al. (Wang et al. 2014), whole-cell biocatalysis achieved complete conversion at 1.8 M CESCa but only 50% conversion at 3 M under identical conditions, suggesting that mass-transfer resistance or enzyme inactivation may limit catalytic efficiency at higher substrate load. To overcome these barriers and enhance space–time yield, further process intensification, such as design of fed-batch process, simplified downstream separation of biocatalyst and product, will be crucial to fully realize the economic potential of CESH-based L-TA bioproduction.

**Fig. 1:**
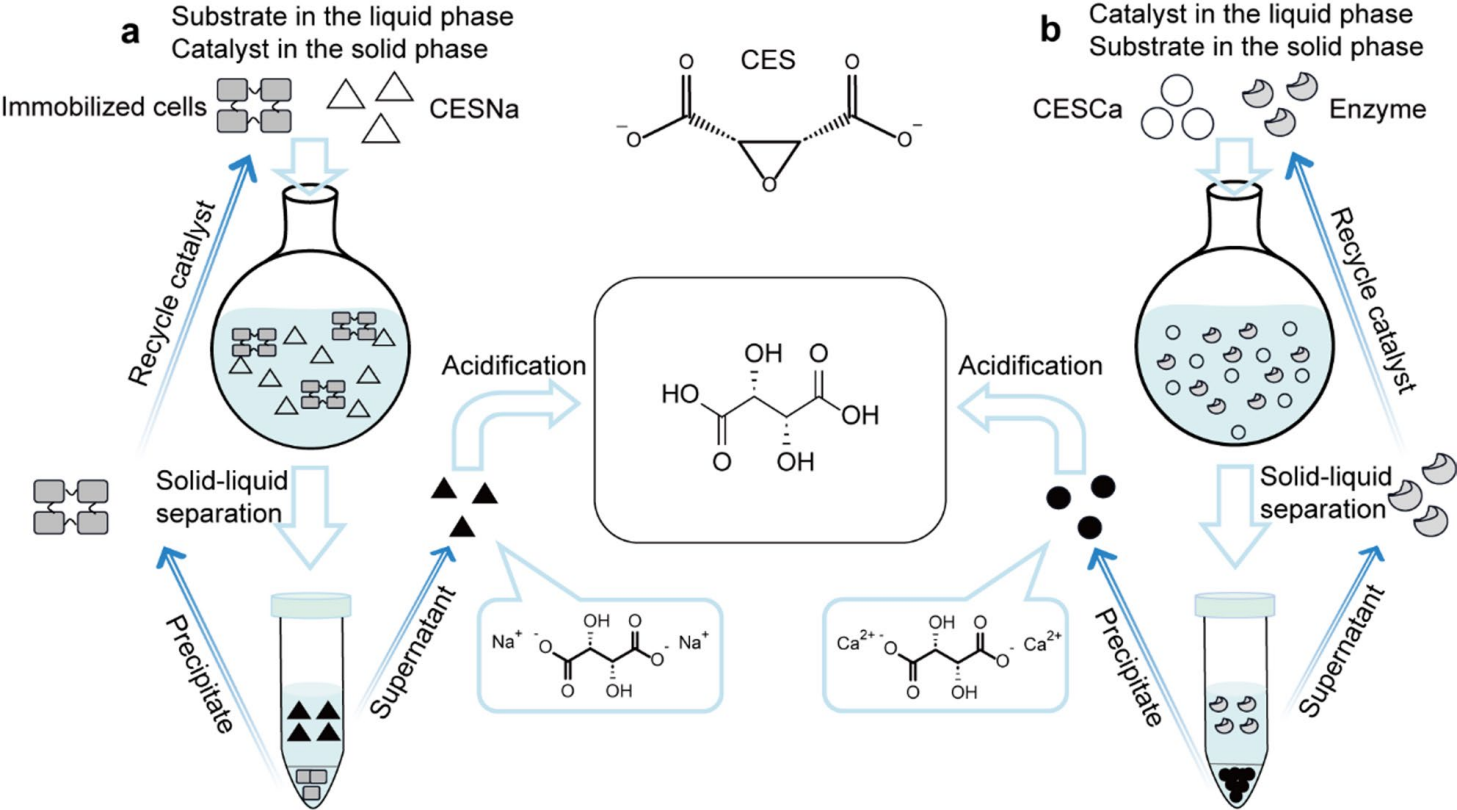
Schematic representation of heterogeneous biocatalytic strategies for the synthesis of L-(+)-tartrate. **a.** Reaction employing cross-linked cell aggregates (CLCAs) as the solid-phase biocatalyst and sodium *cis*-epoxysuccinate (CESNa) as the soluble substrate. **b.** Reaction using crude enzyme as the biocatalyst and poorly soluble calcium *cis*-epoxysuccinate (CESCa) as the substrate

In our previous study, *Bm*CESH was identified from *Bradyrhizobium mercantei* with a high specific activity (550 U/mg) and a low *K*_M_ (5.4 mM) (Han et al. 2024). However, its application potential remained insufficiently characterized. In this study, we established and compared two heterogeneous fed-batch bioconversion systems for the bioproduction of L-TA using *Bm*CESH (Fig. 1). First, cross-linked cell aggregates (CLCAs) containing *Bm*CESH were prepared using low-cost polyethylenimine (PEI) and glutaraldehyde (GA), exhibiting good stability and reusability in preliminary assays. We then applied the CESNa–CLCAs system in fed-batch reactions to confirm its practicality. Furthermore, CESCa-CESH cell-free extract system was optimized via single-factor trials and subsequent fed-batch runs. By removing the mass-transfer constraints of whole-cell catalysts, this approach significantly boosted both total turnover number (TTN) and the space–time yield (STY) of L-TA production.

## Materials and methods

### Reagents

Polyethylenimine (PEI) and glutaraldehyde (GA) were purchased from Shanghai Titan Scientific Co., Ltd. (Shanghai, China). Sodium *cis*-epoxysuccinate and calcium *cis*-epoxysuccinate were prepared following the protocol of Miková et. al (Miková. et al. 1998). The starting materials, including maleic anhydride, sodium hydroxide, calcium carbonate, and sodium tungstate dihydrate, were purchased from Shanghai Macklin Biochemical Technology Co., Ltd. (Shanghai, China). Hydrogen peroxide solution (30% w/w) was obtained from Sinopharm Chemical Reagent Co., Ltd. (Shanghai, China). TieChui *E. coli* Lysis Buffer was purchased from Shanghai Chuzhi Biotechnology Co., Ltd. (Shanghai, China). PBS (phosphate-buffered saline) was prepared as follows: 137 mM NaCl, 1.07 mM KCl, 4.05 mM Na_2_HPO_4_, 0.72 mM KH_2_PO_4_ (pH 7.4).

### *Bm*CESH expression and cell lysis

The codon-optimized *Bm*CESH gene from *Bradyrhizobium mercantei* (GenBank ID: WP_076865492.1) was expressed using the recombinant *E. coli* BL21(DE3)/pET-28a( +) system, as previously constructed in our laboratory (Han et al. 2024). The recombinant *E. coli* strain was cultured in 1 L of Terrific Broth medium containing (per liter): 12 g tryptone, 24 g yeast extract, 16.43 g K_2_HPO_4_·3H_2_O, 2.31 g KH_2_PO_4_, and 5 g glycerol. Cells were grown at 37 °C and 220 rpm until mid-log phase (OD_600_ = 0.6–0.8). Expression of protein was then induced by adding lactose to a final concentration of 0.1% (w/v), followed by continued incubation at 25 °C and 220 rpm for 35 h. Following cultivation, the bacterial culture was transferred to centrifuge bottles and pelleted by centrifugation at 8,000 × *g* for 40 min at 4 °C. The supernatant was carefully decanted, and the cell pellet was gently resuspended in ice-cold PBS (pH 7.4) for washing. After repeating the centrifugation under identical conditions, the washed cell pellet was aliquoted into sterile microtubes and immediately stored at –20 °C for subsequent use.

To prepare the CESH cell-free extract for activity assays, 1 g (wet weight) of cell pellet was resuspended in 9 mL of ice-cold PBS to obtain a 10% (w/v) cell suspension. Subsequently, 1 mL of TieChui *E. coli* Lysis Buffer was added to achieve a final volume of 10 mL, corresponding to a 1:10 buffer-to-biomass ratio. The mixture was incubated at 4 °C for 20 min with gentle agitation to ensure complete cell lysis. The crude lysate was centrifuged at 12,000 × *g* for 15 min at 4 °C, and the supernatant was collected as the CESH cell-free extract.

### Characterization of specific activity

Whole-cell suspensions or cell-free extract were appropriately diluted with PBS (pH 7.4) to achieve a standardized cell-equivalent concentration corresponding to 0.8 mg/mL of wet cell weight, which served as the biocatalyst stock solution for subsequent activity assays.

For the activity assay, each reaction was conducted in a 200 μL mixture containing 160 μL of assay buffer (PBS supplemented with 0.01% (v/v) Tween-20), 20 μL of the biocatalyst stock solution, and 20 μL of 200 mM sodium *cis*-epoxysuccinate. The mixture was incubated at 37 °C with continuous shaking at 1000 rpm for 5 min in a temperature-controlled shaking incubator. The reaction was then quenched by the addition of 100 μL of 1 M sulfuric acid.

L-( +)-Tartaric acid was quantified by high-performance liquid chromatography (HPLC) using an Agilent 1260 Infinity II system equipped with a Betasil™ C18 reversed-phase column (5 μm, 4.6 mm × 250 mm; Thermo Fisher) and a UV detector set at 210 nm. Chromatographic separation was performed at a flow rate of 1.0 mL/min using a mobile phase consisting of 0.02 M potassium dihydrogen phosphate buffer (pH adjusted to 2.1–2.5 with phosphoric acid; 95% v/v) and acetonitrile (5% v/v). The column temperature was maintained at 30 °C, and 10 μL of each sample was injected. Quantification was performed using external calibration with L-( +)-tartaric acid standards.

Prior to HPLC analysis, all samples were centrifuged at 12,000 × *g* for 10 min to remove particulates, and the resulting supernatants were used for analysis. One unit of enzyme activity (1 U) was defined as the amount of enzyme required to convert 1 μmol of *cis*-epoxysuccinate per minute under the specified assay conditions.

### Preparation of cross-linked cell aggregates

Cross-linked cell aggregates (CLCAs) were prepared in two steps: (i) flocculation of cells using PEI (pH adjusted to 7.0 with 1 M HCl), and (ii) cross-linking with GA. To evaluate the effect of PEI concentration on whole-cell activity, a 10% (w/v) PEI solution was incrementally added (10, 10, 30, 50, 100, and 4 × 200 μL) to 10 mL of a cell suspension (100 mg/mL in PBS, pH 7.4). After each addition, the mixture was gently stirred for 30 min, and a 100 μL aliquot was withdrawn for activity measurement.

Subsequently, CLCAs were prepared by adding 10% (w/v) GA to 1 mL of the cell–PEI co-aggregates to achieve final GA concentrations of 0.4% (w/v), followed by continuous stirring at 10 rpm for 30 min at room temperature. The resulting CLCAs were collected by centrifugation (5,000 × *g*, 10 min), washed with PBS (pH 7.4) to remove unreacted glutaraldehyde, and resuspended in an equal volume of PBS for activity analysis.

### Characterization of CLCAs

The thermal stability of CLCAs was assessed by measuring the bioconversion efficiency of 1 M CESNa (dissolved in Milli-Q water and adjusted to pH 7.0 with NaOH) at 30 °C, 40 °C, and 50 °C. pH stability was evaluated by determining the bioconversion efficiency of 1 M CESNa at 37 °C in reaction mixtures with pH values ranging from 5.0 to 10.0, adjusted using 0.1 M HCl or 0.1 M NaOH. To investigate storage stability, both CLCAs and free cells were stored at room temperature (25 ± 2 °C) for 133 days, with periodic sampling to monitor changes in specific activity over time. Substrate tolerance of CLCAs was examined by incubating CLCAs (with a CESH dosage of 17.5 kU/L) in 1–3 M CESNa solutions at 37 °C, and monitoring the reaction progress over a 5-h period.

### Batch and fed-batch bioconversion

The reusability of CLCAs was evaluated through five consecutive batch bioconversion cycles. CLCAs, containing CESH at a dosage of 60 kU/L, were added to 10 mL of 1 M CESNa (pH 7.0) and incubated at 37 °C with shaking at 300 rpm. Upon complete conversion of CESNa, the CLCAs were recovered by centrifugation (5,000 rpm, 10 min, 4 °C), and then resuspended in fresh 10 mL of 1 M CESNa for the subsequent cycle.

Based on the results of the batch bioconversion process, a fed-batch strategy was designed. To maintain consistent reaction performance, CLCAs (containing CESH at 12 kU/L) were supplemented at the beginning of each cycle following the initial reaction. The TTN of CLCAs was calculated indirectly based on activity loading, serving as a metric to evaluate operational and process stability. The formulae could be written as Eq. (1):

1 $$ {\mathrm{TTN}} = \frac{{{\mathrm{n}}_{{\mathrm{s}}} }}{{{\mathrm{n}}_{{\mathrm{E}}} }} = \frac{{{\mathrm{n}}_{{\mathrm{s}}} \times {\mathrm{A}}_{{{\mathrm{pure}}}} \times {\mathrm{M}}_{{\mathrm{r}}} }}{{\mathrm{[E]}}} $$

where *n*_*s*_ is moles of substrate converted, mol; *n*_*E*_ is moles of enzyme consumed, mol; *A*_*pure*_ is pure enzyme’s specific activity, U/g; *M*_*r*_ is pure enzyme’s relative molecular mass, g/mol; [*E*] is total enzyme activity loading, U.

### CESCa reaction optimization

Bioconversion reactions were carried out using CESCa as the substrate at concentrations ranging from 1.0 to 3.0 M, where it was not fully soluble, with crude *Bm*CESH cell-free extract applied as the biocatalyst at a final activity of 17.5 kU/L. Reactions were performed at 37 °C with continuous shaking at 300 rpm in an incubator to ensure uniform mixing of the turbid suspension. At designated time points, 20 μL aliquots were withdrawn and immediately quenched by addition to 80 μL of 1 M HCl. The mixture was thoroughly vortexed and subsequently diluted tenfold with Milli-Q water. A 10 μL portion of the diluted sample was then subjected to HPLC analysis.

To evaluate the reusability of the crude *Bm*CESH enzyme, fed-batch bioconversion of CESCa was conducted. When the reaction rate declined, additional cell-free extract of 10 kU/L was supplemented to sustain high catalytic efficiency.

## Results and discussion

### Optimization of CLCAs preparation

**Fig. 2:**
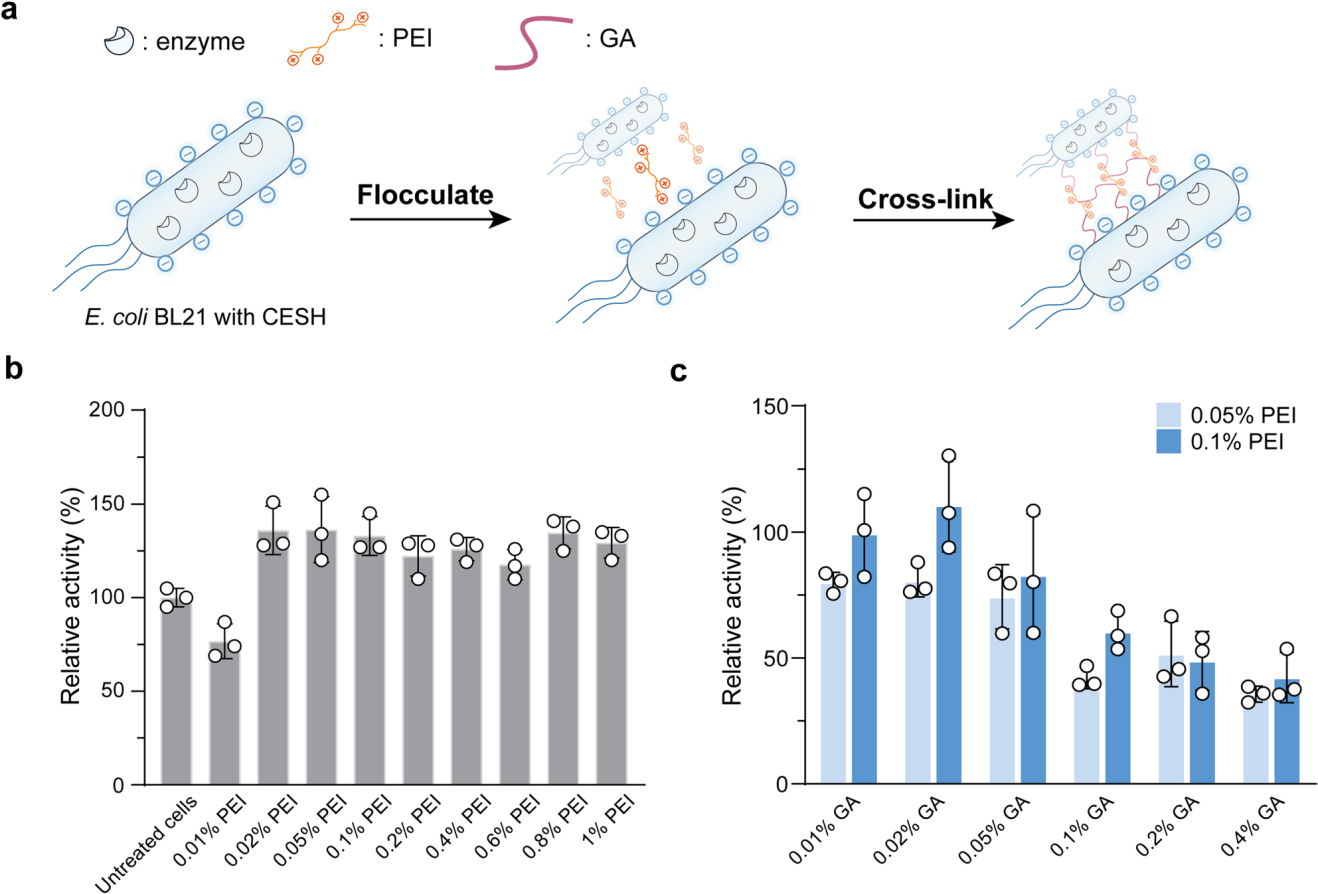
Optimization of CLCAs preparation. **a** Schematic workflow for the preparation of CLCAs. **b** Effect of polyethylenimine (PEI) concentration on the relative activity of cell aggregates. The activity of untreated cells was set as 100%. **c** Effect of glutaraldehyde (GA) concentration on the activity recovery of CLCAs during cross-linking of PEI- cell aggregates. The activity of untreated cells was set as 100%. Data in the figure are shown as the mean ± SD of three replicates

PEI, a commonly used electrostatic flocculating agent, facilitates rapid cell aggregation and sedimentation at low concentrations. Branched PEI contains abundant cationic amino groups on its surface, which strongly interact with the anionic phospholipid bilayer of *E. coli* cells via electrostatic binding (Fig. 2a). As shown in Fig. 2b, the activity recovery of cell aggregates decreased slightly at a PEI concentration of 0.01% (w/v). Interestingly, when the PEI concentration increased to the range of 0.02%–1.00% (w/v), the relative activity exceeded 100%. Further increases in PEI concentration beyond 0.1% did not significantly alter relative activity. These results suggest that PEI at concentrations ≥ 0.02% (w/v) induced sufficient cell aggregation and enhanced apparent activity, potentially due to increased membrane permeability resulting from electrostatic interactions between PEI and cell surface phospholipids. Such interactions may facilitate substrate uptake and product release, thereby improving catalytic efficiency (Soh et al. 2020).

The concentration of GA plays a critical role in determining the catalytic activity of CLCAs. At suboptimal GA concentrations, cross-linking efficiency is reduced, leading to decreased structural stability of the aggregates (Zaak et al. 2017). In contrast, excessive GA can result in non-specific reactions with amino groups near enzyme active sites, thereby diminishing catalytic activity. As shown in Fig. 2c, increasing the GA concentration from 0.01% to 0.02% (w/v) led to a rise in relative activity, reaching a maximum of 111%. However, further increases in GA concentration up to 0.40% resulted in a sharp decline in relative activity to 30–40%, likely due to over-crosslinking and active-site modification. In addition, among the PEI-crosslinked aggregates, CLCAs prepared with 0.10% PEI exhibited higher catalytic activity than those crosslinked with 0.05% PEI, under identical GA treatment. Based on these findings, the optimal formulation for CLCAs preparation was determined to be 0.10% PEI in flocculation combined with 0.02% GA in cross-linking.

### Batched biotransformations of CESNa using CLCAs at a 10 mL scale

**Fig. 3:**
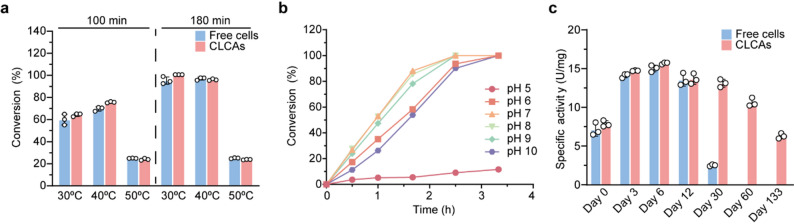
Characterization of CLCAs. **a** Operational stability of free cells and CLCAs at 30 °C, 40 °C and 50 °C. Data in the figure are shown as the mean ± SD of three replicates. **b** Reaction profile of CLCAs-catalyzed hydrolysis of 1.0 M CESNa across a pH range of 5–10. The experiment was performed in a single replicate. **c** Storage stability of free cells and CLCAs over a 133-day period at room temperature. Data in the figure are shown as the mean ± SD of three replicates

The catalytic performance of CLCAs was evaluated under varying reaction temperatures, pH conditions, and storage durations (Fig. 3). As shown in Fig. 3a, the CLCAs exhibited slightly better tolerance at 40 °C compared to free cells, and their catalytic performance at this temperature was superior to that at 30 °C. However, at 50 °C, the conversion rates of both free cells and CLCAs declined significantly. The identical conversion values at 100 min and 180 min suggest that both free cells and CLCAs completely lost their catalytic activity after 100 min of incubation at 50 °C. These results indicate that, although CLCAs offer the advantage of easier separation from the product during CESNa biotransformation, they provide only limited improvement in thermostability.

To evaluate the pH tolerance of CLCAs, CESNa bioconversion was performed under various pH conditions, and the reaction progress curves were plotted accordingly. As shown in Fig. 3b, the CLCAs exhibited the highest catalytic activity at pH 7–9, moderate activity at pH 6 and 10, and poor activity at pH 5. The reduced activity under acidic condition was likely due to the protonation of the catalytic Asp residue, as previously reported (Dong et al. 2024; Han et al. 2024). By comparing the catalytic rates of CLCAs during the 2–3 h biotransformation period, we observed that the catalytic rates (slope of curves in Fig. 3b) remained steady across all tested pH conditions until reaching approximately 90% of conversion. These results indicate that the CLCAs possess broad pH tolerance, with an optimal activity at pH 7.0, which was selected for subsequent experiments.

Subsequently, the storage stability of both free cells and CLCAs was evaluated by monitoring their specific activity over a period of 133 days under identical preservation conditions at room temperature. As shown in Fig. 3c, during the first three days of storage, the activity of both free cells and CLCAs increased markedly by 87%, likely due to enhanced cell membrane permeability and improved substrate mass transfer. After 30 days, the activity of free cells had declined by 82% compared to day 3, whereas the CLCAs retained 89% of their day 3 activity. Notably, the CLCAs maintained 40% of their peak activity even after 133 days of storage, demonstrating significantly improved long-term storage stability.

### Performance of CLCAs in batch and fed-batch reactions

**Fig. 4:**
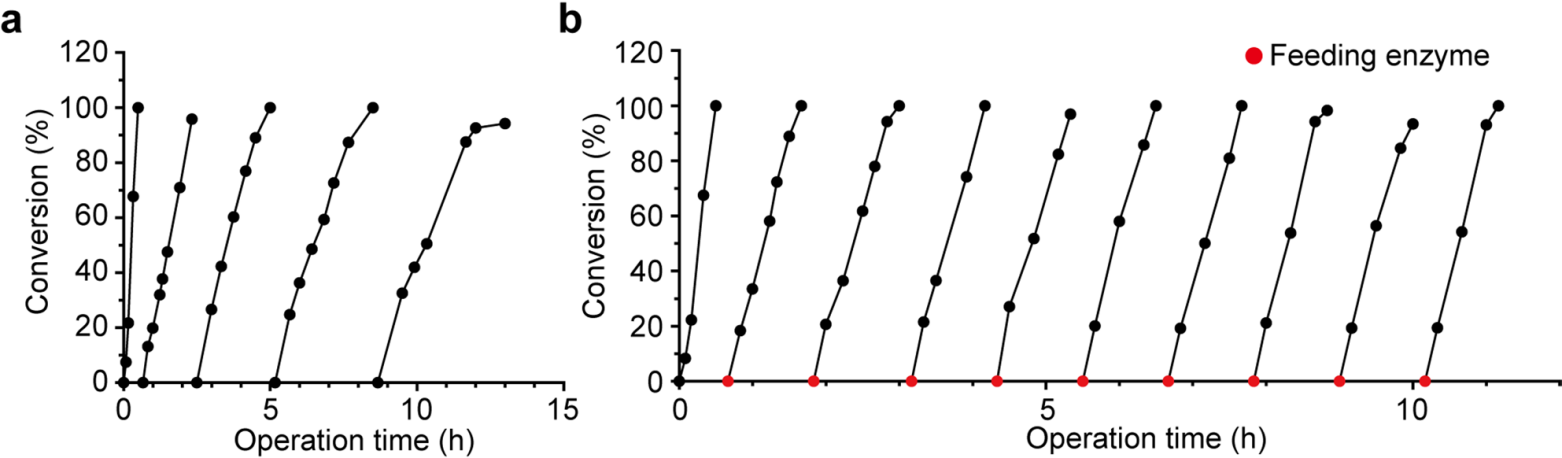
Batched biotransformations of 1.0 M CESNa using CLCAs at a 10 mL scale. **a** Reaction progress using CLCAs at an activity dosage of 60 kU/L. **b** Fed-batch reaction using an initial dose of 60 kU/L CLCAs, followed by supplementation with 12 kU/L per batch. The processing time between reaction cycles was 10 min on average. Reactions were stopped as soon as the conversion reached 95%. On average, the interval between consecutive reaction cycles was 10 min

The application of immobilized cells in biotransformation offers dual advantages including simplified downstream processing and improved cost-effectiveness through catalyst reusability. To assess their performance, CLCAs were subjected to five consecutive reaction batches, each replenished with 1.0 M substrate (Fig. 4a). In the initial batch, 60 kU/L of CLCAs were loaded, achieving complete conversion within 30 min. In subsequent batches, progressively longer reaction times were required to reach full conversion. By the fifth batch, the reaction reached only 92% conversion after 260 min, indicating that approximately 10% of the original catalytic activity remained. The decline in conversion efficiency was likely due to mechanical damage to the CLCAs, enzyme leaching, and enzyme deactivation. The average loss of enzymatic activity per cycle was approximately 20% of the initial load (60 kU/L). Despite the gradual activity loss, the average space–time yield over the first four cycles was maintained at 60.8 g L^−1^ h^−1^ (based on L-TA as the product, molecular weight 150 Da). The TTN of CLCAs reached 1.7 × 10^6^ (calculated based on the specific activity of *Bm*CESH, 550 U/mg), exceeding the industrial benchmark (TTN > 10^5^) by over an order of magnitude. These results highlight the strong potential of CLCAs for industrial-scale biotransformation processes.

Based on the observed activity loss of CLCAs in five consecutive batch reactions, a fed-batch strategy was developed to maintain reaction stability. In this approach, fresh CLCAs (12 kU/L) were added to compensate for the loss of CESH activity (Fig. 4b). With this supplementation, each reaction batch was completed within an average of 70 min, and no significant prolongation in reaction time was observed as the number of batches increased. This fed-batch strategy effectively maintained consistent reaction performance while enhancing operational stability. Compared to the conventional batch process, it achieved a space–time yield of 150 g L^−1^ h^−1^, representing a substantial enhancement in overall productivity.

**Fig. 5:**
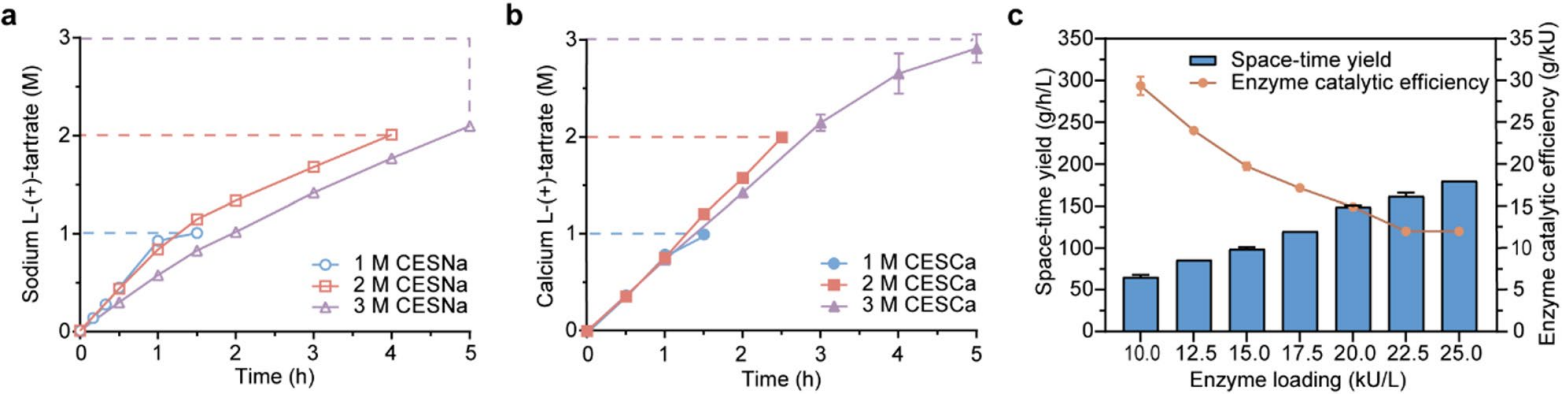
Optimization of CES bioconversion conditions to improve space–time yield. **a** CLCAs-mediated bioconversion of CESNa at substrate concentrations of 1.0–3.0 M using an activity dosage of 17.5 kU/L in 10 mL reaction volume at 37 °C. **b** Bioconversion of 1.0–3.0 M CESCa using CESH crude enzyme at an activity dosage of 17.5 kU/L in 10 mL substrate solution at 37 °C. **c** Effect of biocatalyst dosage (10–25 kU/L) on catalytic efficiency and space–time yield in a 2 M CESCa bioconversion system using CESH crude enzyme. Data are presented as mean ± SD from three independent replicates

To further enhance CESH-mediated biosynthesis of L-tartrate, reactions with higher substrate loading were investigated. Substrate concentrations ranging from 1 to 3 M were evaluated in the CESNa–CLCAs reaction system, with reaction progress monitored over a 5-h period (Fig. 5a). The reaction profiles showed comparable initial catalytic efficiencies between the 1.0 M and 2.0 M substrate concentrations. However, after 1 h of reaction, the catalytic rate of the 2.0 M condition decreased significantly, requiring 4 h to complete the reaction. In addition, the 3.0 M condition exhibited lower catalytic efficiency even in the early stage (within 30 min), followed by more rapid activity decay over time, resulting in less than 70% conversion after 5 h. This concentration-dependent activity attenuation suggests that excessive substrate concentrations may accelerate the inactivation of *Bm*CESH, with the early-stage efficiency suppression especially observed in the 3.0 M condition.

### CESCa-CESH heterogeneous biocatalysis

To overcome the limitations of CESH inactivation under high substrate loading while maintaining a high space–time yield, an alternative heterogeneous reaction system was developed using CESCa, the poorly soluble salt form of the substrate, and *Bm*CESH cell-free extract as the biocatalyst. To ensure efficient conversion of CESCa, both substrate concentration and enzyme loading were systematically optimized (Fig. 5). Initial experiments were conducted with 1.0 M to 3.0 M substrate concentrations to monitor the bioconversion process (Fig. 5b). The catalytic rate remained largely unaffected across all concentrations, with only a slight decline observed at 3.0 M once conversion exceeded 80%. These results demonstrate significantly improved tolerance to high substrate loading compared to the CESNa–CLCAs system. This advantage is likely attributable to the limited solubility of CESCa at 37 °C (10 g/L *vs.* 735 g/L for CESNa) (Wang et al. 2014), which maintains the substrate concentration in solution below the threshold that induces enzyme inactivation. Meanwhile, the low *K*_M_ (5.0 mM) of *Bm*CESH ensures near-maximal catalytic efficiency under both poorly soluble CESCa (58.8 mM in slurry) and highly soluble CESNa (3.0 M) conditions. Additionally, the product calcium tartrate has over fivefold lower solubility than CESCa, promoting in situ precipitation of the product. This not only drives the reaction equilibrium forward but also facilitates downstream processing. Together, these findings highlight the CESCa-CESH system’s superior suitability for high-substrate-loading bioconversions. Further optimization of enzyme loading was conducted to improve process performance. As shown in Fig. 5c, the space–time yield of L-tartrate increased proportionally with enzyme loading, indicating the system’s strong potential for high-productivity industrial applications.

### Fed-batch reactions of CESCa-CESH system

**Fig. 6:**
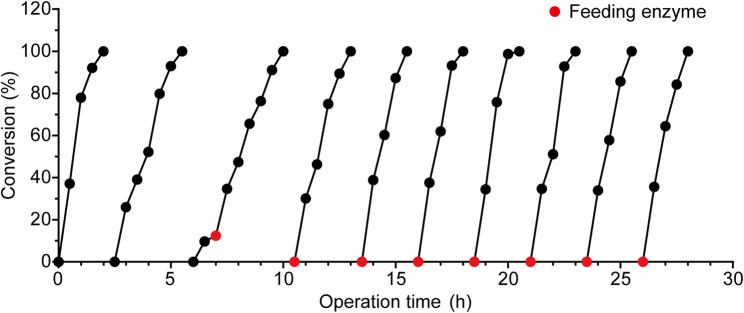
Fed-batch bioconversion of 2.0 M CESCa (10 mL reaction volume) using crude CESH enzyme. The reaction was initiated with an enzyme activity loading of 20 kU/L, followed by supplementation with 10 kU/L starting from the third batch. Fresh substrate (2.0 M CESCa) was replenished in each batch. On average, the interval between consecutive reaction cycles was 30 min

To improve the utilization of enzymatic activity, a fed-batch reaction strategy was developed for the CESCa-CESH system (Fig. 6). The initial dosage of CESH was 20 kU/L for the biotransformation of 2.0 M CESCa. Following each reaction cycle, the residual enzyme in the supernatant was recovered by centrifugation and reused in subsequent cycles. A markedly prolonged conversion time was observed in the second reaction cycle compared with the first cycle. The residual activities measured in the supernatant were 72 ± 2% after the first cycle and 29 ± 2% after the second. This decline may result from enzyme inactivation during the reaction or from adsorption/encapsulation of *Bm*CESH by the calcium tartrate crystals. Therefore, an additional 10 kU/L of CESH was supplemented from the third cycle onward. The conversion time per cycle was maintained at 2 h, comparable to that of the first cycle, thereby improving overall enzyme utilization efficiency. Under these conditions, the system achieved a TTN of 3.3 × 10^6^, substantially outperforming the CESNa–CLCAs system (TTN = 1.7 × 10^6^). Moreover, the optimized fed-batch strategy reached the space–time yield of 136 g L^−1^ h^−1^ as well as a high specific productivity of 27.3 g/kU (484 g_product_/g_catalyst_, Table 1).

**Table 1:** Comparison of reported reaction systems for L-TA bioconversion

Enzyme	*Bm*CESH	*Bm*CESH	*Bm*CESH	*Ro*CESH	*Labrys* sp. BK-8 CESH
Catalyst format	CLCAs	Cell free extract	Whole-cell	Whole-cell	Immobilized cell
Substrate	CESNa	CESCa	CESNa	CESNa/CESCa^a^	CESNa
Biocatalyst loading (g/L)	1.1^b^	0.62^b^	2.9	0.35	50^b^
Substrate loading (M)	1	2	1	3	1
Enzyme activity loading (kU/L)^c^	16.8	11	n.a	2.4	170
Conversion time (h)^d^	0.97	2.2	2.7	20	10
Specific productivity (g/kU)	8.9	27.3	n.a	187.5	0.9
Specific productivity (g_product_/g_catalyst_)	136	484	52	1286	3
Space–time yield (g L^−1^ h^−1^)	150	136	55.6	22.5	15.0
Reference	This study	This study	(Han et al. 2024)	(Wang et al. 2014)	(Bao et al. 2015)

^a^A mixture containing 1.8 M CESCa and 1.2 M CESNa was used for the biotransformation. ^b^Biocatalyst loading is based on the wet cell weight. ^c^For multi-cycle reactions, the enzyme activity shown represents the average loading per cycle. ^d^For multi-cycle reactions, the reaction time shown represents the average duration per cycle. ^e^n.a., not available

Compared to conventional *Bm*CESH whole-cell catalysis, the present study significantly enhanced the enzyme’s industrial applicability by reducing the cellular biomass requirement to 21% of the original quantity while achieving a 2.5-fold improvement in space–time yield (Han et al. 2024). These results collectively demonstrate that the optimized fed-batch CESCa-CESH system offers significantly enhanced catalytic efficiency and productivity, underscoring its strong potential for industrial-scale biocatalytic applications. Economically, the process offers a clear advantage because, unlike the CESNa-based route, the CESCa-based synthesis eliminates the need for 2.5 equivalents of NaOH (2 CNY/kg). In both cases CaCO_3_ is required for substrate or product precipitation, so the only net change in reagent consumption is the reduced use of NaOH. This reduction leads to a net cost decrease of ~ 40%, taking the cost of maleic anhydride (5 CNY/kg) as the 100% reference per batch (Miková. et al. 1998).

## Conclusions

Traditional production of L-tartaric acid uses *cis*-epoxysuccinate hydrolase (CESH) to hydrolyze sodium *cis*-epoxysuccinate (CESNa) into sodium L-tartrate, which is then precipitated by calcium ion addition and acidified to give L-tartaric acid. In this study, we demonstrated two complementary strategies to simplify the recovery of enzyme from the product through solid–liquid separation.

In Strategy A, whole-cell biocatalysts were immobilized (as cross-linked cell aggregates, CLCAs) enabled efficient CESNa hydrolysis with easy catalyst recovery, while Strategy B utilized poorly soluble CESCa substrate where self-limited dissolution prevented enzyme inactivation and facilitated in-situ calcium tartrate precipitation. Fed-batch operation enhanced both systems by maintaining enzymes near *V*_max_, boosting productivity with space–time yields reaching 136 g L^−1^ h^−1^ and specific productivity of 484 g_product_/g_catalyst_ in analogous systems.

Overall, the optimized fed-batch process significantly improves the productivity, operational simplicity, and cost efficiency of the CESCa–CESH system, highlighting its strong potential for industrial implementation. Future efforts should focus on addressing the remaining challenges, including developing continuous-flow operations to further simplify the biotransformation process and enhance space–time yield, as well as improving enzyme stability and cycle-to-cycle recoverability to reduce overall biocatalyst costs. We anticipate that such a flow-integrated system would simplify reactor operation and downstream purification, enabling truly continuous, intensified production of L-TA.

## Data Availability

The datasets used and/or analysed during the current study are available from the corresponding author on reasonable request.

## Abbreviations

CES: *cis*-Epoxysuccinate
CESHs: *cis*-Epoxysuccinate hydrolases
CESNa: Sodium *cis*-epoxysuccinate
CESCa: Calcium *cis*-epoxysuccinate
L-TA: L-(+)-Tartaric acid
TTN: Total turnover number
CLCAs: Cross-linked cell aggregates
PEI: Polyethylenimine
GA: Glutaraldehyde
